# Prevalence and predictors of post-COVID-19-related symptoms: an extended follow-up among employees in health and welfare services in Germany: a short report

**DOI:** 10.3205/dgkh000577

**Published:** 2025-08-29

**Authors:** Peter Koch, Lara Steinke, Claudia Peters, Albert Nienhaus

**Affiliations:** 1 Competence Center for Epidemiology and Health Services Research for Healthcare Professionals (CVcare), Institute for Health Services Research in Dermatology and Nursing (IVDP), University Medical Center Hamburg-Eppendorf (UKE), Hamburg, Germany; 2 University of Lübeck, Lübeck, Germany; 3 Department for Occupational Medicine, Hazardous Substances and Health Sciences (AGG), Institution for Statutory Accident Insurance in the Health and Welfare Services (BGW), Hamburg, Germany

**Keywords:** COVID-19, post-acute COVID-19 syndrome, post-COVID-19 syndrome, health personnel, social workers, persistent symptoms, follow-up, time to symptom-free

## Abstract

**Objective:**

To describe persistent symptoms after a work-related COVID-19 infection in health and welfare workers and the identification of predictors of these symptoms.

**Methods:**

This short report summarises updated results on a bidirectional cohort study of employees in the health and welfare services who had reported a work-related SARS-CoV-2 infection in 2020. Participants were interviewed for the fourth time (T4) in April 2023 using a paper-and-pencil questionnaire. In this extended follow-up study (total prospective follow-up time: 26 months, maximum observation time 32 months), questions were asked about the type and severity of persistent symptoms. Kaplan-Meier curves were used to visualize cumulative survival rates, and Cox regression was used to identify predictors.

**Results:**

Of the 2,053 participants in the baseline study (response rate: 47%), 1,075 people took part in the 4^th^ survey (follow-up rate: 52%); the analysis sample for the longitudinal study comprised 1,809 participants. The most frequently reported persistent symptoms at T4 were fatigue (61%), concentration or memory problems (55%) and shortness of breath (49%). After 12 weeks, the cumulative survival rate was 76.3%, after 12 months 69.3%, and after 32 months 60.0%. Female gender was a statistically significant risk factor for a longer recovery time (Hazard Ratio [HR]: 0.8, 95% CI: 0.63–0.93, p=0.007) as was older age (HR ≥50 years 0.6, 95% CI: 0.51–0.76, p<0.001). Participants with one pre-existing condition had a 20% statistically significant increased risk (HR: 0.8, 95% CI: 0.66-0.95, p= 0.010), subjects with two pre-existing conditions a HR of 0.6 (95% CI: 0.46–0.75, p<0.001) and those with ≥3 pre-existing conditions had a HR of 0.3 (95%-CI: 0.23–0.48, p<0.001). Risk increases were also observed for the number of severe acute symptoms: the more symptoms, the greater the increase in risk. Individuals with medical activity (physicians) were 50% less likely to have a longer time to recovery compared to all other occupational groups (HR: 1.5, 95%-CI: 1.21–1.89, p<0.001).

**Conclusion:**

Minimal further recovery was observed in this cohort of insured persons after a 26-month prospective follow-up. Identified risk factors for persistent symptoms, i.e. female gender, older age, severe acute symptoms, and pre-existing illnesses, define a high-risk group of individuals, who should receive sufficient attention in the early phase of their COVID-19 disease and receive appropriate therapy to minimize the risk of post-COVID-19 syndrome.

## Zusammenfassung

**Zielsetzung:**

Untersuchungsgegenstand der vorliegenden Studie war die Beschreibung persistierender Symptome nach einer arbeitsbezogenen SARS-CoV-2-Infektion bei Beschäftigten im Gesundheitsdienst- und der Wohlfahrtspflege sowie die Identifizierung von Prädiktoren für persistierende Symptome.

**Methoden:**

Der vorliegende Short Report fasst die aktualisierten Ergebnisse einer bidirektionalen Kohortenstudie bei Beschäftigten im Gesundheitsdienst und der Wohlfahrtspflege zusammen, die im Jahr 2020 eine arbeitsbedingte SARS-CoV-2-Infektion gemeldet hatten. Die Teilnehmenden wurden im April 2023 zum vierten Mal anhand eines Papierfragebogen befragt. Bei dem vorliegenden erweiterten Follow-up (prospektive Follow-up-Zeit insgesamt: 26 Monate, maximale Beobachtungszeit: 32 Monate) wurden Fragen zu Art und Schweregrad von persistierenden Symptome gestellt. Zur Darstellung der kumulativen Überlebensraten wurden Kaplan-Meier-Kurven verwendet, und zur Ermittlung der Prädiktoren wurde Cox-Regression gerechnet.

**Ergebnisse:**

Von den 2.053 Teilnehmenden der Baseline-Befragung (Response Rate: 47%) nahmen 1.075 Personen an der 4. Erhebung teil (Follow-up-Rate: 52%); das Analysesample für die Längsschnittstudie umfasste 1.809 Teilnehmende. Die am häufigsten angegebenen persistierenden Symptome zum Zeitpunkt T4 waren Müdigkeit (61%), Konzentrations- oder Gedächtnisprobleme (55%) und Kurzatmigkeit (49%). Nach 12 Wochen betrug die kumulative Überlebensrate 76,3%, nach 12 Monaten 69,3% und nach 32 Monaten 60,0%. Weibliches Geschlecht war ein statistisch signifikanter Risikofaktor für eine längere Zeit bis zur Genesung (HR: 0,8 95%-CI: 0,63–0,93, p=0,007) sowie ein höheres Alter (HR ≥50 Jahre: 0,6 95%-CI: 0,51–0,76, p<0,001). Teilnehmende mit einer Vorerkrankung hatten ein um 20% statistisch signifikant erhöhtes Risiko (HR: 0,8 95%-CI: 0,66–0,95, p= 0,010), mit zwei Vorerkrankungen eine Risikoerhöhung von 0,6 (95%-CI: 0,46–0,75, p<0,001) und diejenigen mit ≥3 Vorerkrankungen hatten eine Hazard Ratio von 0,3 (95%-CI: 0,23–0,48, p<0,001). Eine Risikoerhöhung wurde auch für die Anzahl der schweren akuten Symptome beobachtet: je mehr Symptome, desto größer die Risikoerhöhung. Bei Personen mit ärztlicher Tätigkeit war die Wahrscheinlichkeit einer längeren Erholungszeit im Vergleich zu allen anderen Berufsgruppen um 50% geringer (HR: 1,5 95%-CI: 1,21–1,89, p<0,001).

**Schlussfolgerung:**

In dieser Kohorte von Versicherten wurde nach einer 26-monatigen Nachbeobachtungszeit nur eine minimale weitere Genesung bzgl. persistierender Symptome beobachtet. Die identifizierten Risikofaktoren für persistierende Symptome wie weibliches Geschlecht, höheres Alter, schwere akute Symptome und Vorerkrankungen definieren eine Hochrisikogruppe von Personen, die in der frühen Phase ihrer COVID-19-Erkrankung ausreichend Aufmerksamkeit erhalten und eine angemessene Therapie erhalten sollten, um das Risiko einer Post-COVID-19-Erkrankung zu minimieren.

## Introduction

During the COVID-19 pandemic, many occupational groups started working from home in order to isolate themselves from COVID-19 infection. In contrast, healthcare workers were exposed to a higher infection risk while caring for patients suffering from COVID-19. Therefore, healthcare workers had higher infection rates with Severe Acute Respiratory Syndrome Coronavirus Type 2 (SARS-COV-2) than did other professions [1], [2]. An analysis of German health insurance data found that employees working in healthcare positions had a 2.4-times higher incidence ratio of taking sick leave or being hospitalized due to COVID-19 infection in comparison to workers in other branches [3]. 

It is well known that a SARS-COV-2 infection can result, as in other viral diseases, in symptoms that can persist for a long time [4], [5]. Symptoms persisting longer than four weeks after the initial infection are described as long COVID or post-acute sequelae of SARS-COV-2 (PASC). Symptoms persisting longer than three months that cannot be explained by other causes have been given the name post-COVID-19 syndrome (PCS) [6], [7]. The cardinal symptoms of PCS include fatigue, dyspnoea, taste or smell disorders as well as cognitive impairments that can lead to reduced work ability and quality of life [8], [9].

The estimation of PCS prevalence is still uncertain due to heterogeneous study situation with differences in study population, observation time, study design and PCS definition. With respect to healthcare workers, there are only a few published longitudinal studies with mostly small sample sizes. 

This study aims to estimate the PCS prevalence as a function of the observation time among healthcare workers. In addition, the predictors for the presence of PCS in this population will be identified. The basis of this study is an extended follow-up of a previously published German study.

## Methods

**Figure 1:**
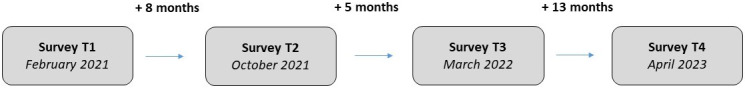
Survey dates of the study

The present exploratory cohort study describes an extended follow-up among insured persons of the German Institution for Statutory Accident Insurance in the Health and Welfare Services (BGW). On the basis of data assessed via three surveys on prevalence and predictors of PCS published by Steinke et al. [10], this short report presents updated results based on the consideration of a further 4^th^ survey. The baseline survey (T1) took place in February 2021, T2 in October 2021, T3 in March 2022, and the T4 survey in April 2023 (Figure 1 (Fig. 1)). The period from the baseline survey to the last survey lasted 26 months. The study was approved by the ethics committee of the Hamburg Medical Association (2021-10463-BO-ff). All participants gave their informed written consent to take part in the study.

### Participants

All insured persons of the BGW of two selected regional administrations in Germany with a work-related SARS-CoV-2 infection in 2020 were invited to take part in the survey (n=4,325). Inclusion criterion was a positive PCR or antigen test, exclusion criteria were the absence of a SARS-COV-2 infection, limited writing and reading skills, and poor German-language skills. Response rate of the baseline survey was 48% (n=2,053), follow-up rates were at T2 70% (n=1,426), T3 63% (n=1,300) and T4 52% (n=1,075). For the survival analysis, 244 participants of the baseline sample were excluded due to asymptomatic SARS-CoV-2 infection, missing test date and missing time to recovery. The final survival analysis sample encompassed 1,809 participants. For the analysis of the symptom progression, only participants who took part in all four surveys and did not have a relapse of symptoms were included in the analysis (n=642).

### Material

The baseline questionnaire assessed sociodemographic information, lifestyle factors (smoking, physical exercise), occupational information and subjective state of health. Furthermore, information on pre-existing diseases and retrospective data on testing date and severity of 13 different symptoms (none/mild/moderate/severe) during the acute phase of the disease were collected. In addition, the presence of persistent symptoms on the day of the survey were collected in the same way as acute symptoms.

The follow-up questionnaires provided further information on the presence of persistent symptoms. If no persistent symptoms were present, time to recovery was assessed in days or weeks in the baseline questionnaire. In the questionnaires at T2 and T3, symptom-free participants could choose between categories “up to 4 weeks”, “up to 3 months”, “up to 6 months”, “up to 12 months” and “longer than 12 months” calculated from the date of test. Time to recovery was not explicitly asked in the T4 questionnaire, but was calculated in another way, as explained in the next subchapter.

### Statistical methods

The statistical procedures are explained in detail at Steinke et al. [10]. A survival analysis was performed, with absence from symptoms representing the event. For participants who reported symptoms again after being symptom-free, only the first event was taken into account. Individual observation time was defined from the date of the test and the date of symptom freedom or the date of censoring. For events reported in the T1 questionnaire, the time interval was specified in the questionnaire. For events in questionnaires T2 and T3, the recovery time was calculated from the mean time of the specified intervals (see above). For events at T4, the recovery time was defined as the mean time between the T3 and T4 survey. A Cox regression was calculated to determine risk factors, and proportional risk assumption was checked using log-minus-log diagrams and time-dependent interaction terms.

For the analysis of symptom progression, only participants for whom data were available at all four time points were included. Statistical differences were calculated using the McNemar test for paired samples, comparing the prevalences of T1 and T4. All significance tests were two-sided with a significance level of 0.05. The analyses were performed with SPSS version 29.

## Results

At time T4, 1075 participants took part in the survey (follow-up rate: 52%). In total, the longitudinal dataset now comprised 1809 participants, which is one person less than in the previous analysis. The description of this cohort can be found in Steinke et al. [10]. 

The T4 dropout analysis showed that younger age, smoking, and no physical exercise were risk factors for dropout.

**Figure 2:**
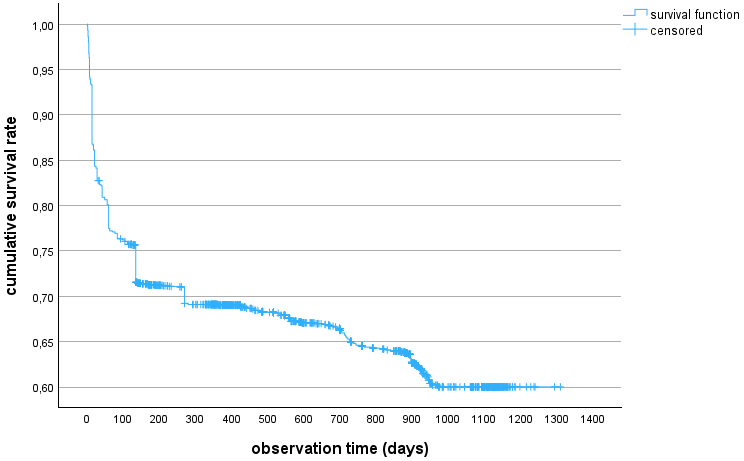
Kaplan-Meier curve of the cohort (n=1,809)

A total of 637 recoveries took place during the observation period. The maximum observation time was 1,310 days; the minimum was 1 day. 25% of the participants experienced recovery by day 135 of their individual observation period. After 12 weeks, the cumulative survival rate was 76.3%, after 12 months 69.3%, and after 32 months 60.0% (Figure 2 (Fig. 2)).

The results of the Cox regression show female gender as a statistically significant risk factor for a longer time to recovery (HR: 0.8, 95% CI: 0.63-0.93, p=0.007) (Table 1 (Tab. 1)). It was also shown that the probability of a longer recovery time increases with increasing age (HR 35–49 years: 0.8, 95% CI: 0.67–1.03, p=0.087 and HR ≥50 years: 0.6, 95% CI: 0.51–0.76, p<0.001). A trend can also be observed for the number of pre-existing conditions: Participants with one pre-existing condition have a 20% statistically significant increased risk compared to those with no pre-existing condition (HR: 0.8, 95% CI: 0.66–0.95, p=0.010), subjects with two pre-existing conditions have a hazard ratio of 0.6 (95%-CI: 0.46–0.75, p<0.001) and those with ≥3 pre-existing conditions have a 70% increased hazard ratio (HR: 0.3, 95%-CI: 0.23–0.48, p<0.001). Risk increases were also observed for participants with 1–2 or with ≥3 severe acute symptoms in comparison with those experiencing no severe acute symptoms (HR: 0.7, 95% CI: 0.61–0.89, p=0.002 and HR: 0.4, 95% CI: 0.34–0.52, p< 0.001). Individuals with medical activity (working as a physician) were 50% less likely to have a longer time to recovery compared to all other occupational groups (HR: 1.5, 95% CI: 1.21–1.89, p<0.001).

In model 2, the number of severe acute symptoms and pre-existing conditions was replaced by the various symptoms and pre-existing conditions (Table 2 (Tab. 2)). The hazard ratios of gender, age, and occupational activity remain unchanged. For cough (HR: 0.7, 95% CI: 0.54–0.96, p=0.027), dyspnea (HR: 0.7, 95% CI: 0.56–0.96, p=0.023), fatigue (HR: 0.6, 95% CI: 0.54–0.78, p<0.001), and concentration or memory problems (HR: 0.6, 95% CI: 0.42–0.75, p<0.001), statistically significant increases in risk were observed. Increases in risk were also observed for cardiovascular diseases (HR: 0.8, 95% CI: 0.62–0.94, p=0.012), respiratory diseases (HR: 0.7, 95% CI: 0.49-0.88, p=0.005) and hormonal/metabolic diseases (HR: 0.7, 95% CI: 0.57–0.88, p=0.002).

**Figure 3:**
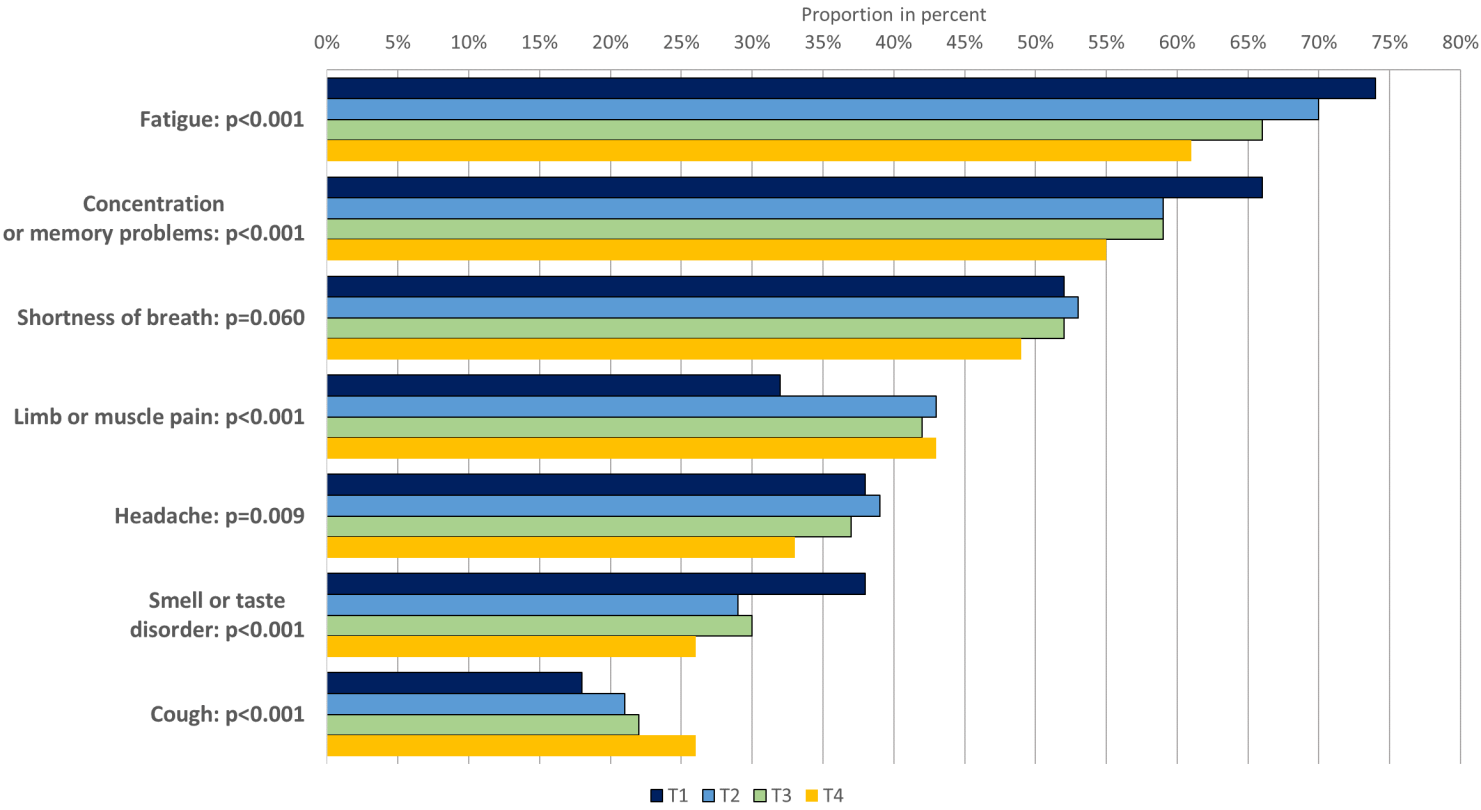
Proportions of selected persistent symptoms in subsample (n=642)

The subsample for analyzing persistent symptoms consisted of 642 subjects who took part in all four surveys, 440 (67%) of whom had persistent symptoms at T4 (Figure 3 (Fig. 3)). The three most frequently mentioned symptoms were fatigue (T4: 61%), concentration or memory problems (T4: 55%), and shortness of breath (49%). For the first two symptoms, a statistically significant decrease in prevalence was observed over time (p<0.001); for shortness of breath, the decrease was not significant (p=0.060). There was also a significant decrease in the prevalence of headaches (T4: 33%, p=0.009) and smell or taste disorder (T4: 26%, p<0.001). There was a statistically significant increase in the prevalence of limb or muscle pain (T4: 42%, p<0.001) and cough (T4: 26%, p<0.001), whereby the limb or muscle pain category at T1 only included limb pain, and from T2 onwards included both pain localisations. 

## Discussion

The present results update and extend the study results of a bidirectional cohort study by Steinke el al. on the basis of a further 13-month follow-up. With regard to persistent symptoms, a cumulative survival rate of 60% was observed at T4 in the cohort of insured persons from the health and welfare services. Risk factors for failure to recover from long COVID-19 symptoms were female gender, older age, number of previous illnesses, and number of severe acute symptoms. Medical activity was observed as a protective factor. The three most frequently mentioned persistent symptoms were fatigue, concentration or memory problems, and shortness of breath, which decreased over time but were still present at a high level at T4 (≥49%).

In comparison to the T3 follow-up results, the cumulative survival rate decreased by 7.2 percentage points, from 67.2% to 60%. This means that in an extended observation period of 13 additional months, 59 more individuals experienced a recovery from PCS, which is 3.3% of the total cohort. The Kaplan-Meier curve illustrates that after 5 months, the largest proportion of symptomatic participants (cumulative survival rate) has fallen to 71%; in the subsequent observation period, this proportion decreases only very slowly. From this data, one would conclude that the probability of recovery for the remaining symptomatic participants is decreasing. Similar courses with an equally rapid decrease in persistent symptoms in the first five months can also be observed in other cohorts [11], [12], [13].

Similar to the T3 analysis, fatigue, concentration or memory problems, and shortness of breath are also the most frequently mentioned persistent symptoms at T4, which is in line with the results of other studies data [14], [15], [16] and have a prevalence of between 49% and 61%. Although a statistically significant reduction in relation to T1 prevalence was observed for the first two symptoms mentioned, the prevalences at the last observation point remain at a high level of over 50%. This also applies to shortness of breath (T4: 49%), even if the reduction is not statistically significant. The statistically significant increase in cough symptoms over time was surprising. A subgroup analysis showed that this increase was even more pronounced in those with pre-existing respiratory illnesses. We are not aware of any data from the literature concerning this observation.

With regard to the predictors of recovery from persistent symptoms, the risk factors identified in the T3 analysis were largely confirmed in the underlying present analysis: the hazard ratios for gender, age, number of pre-existing illnesses, number of severe acute symptoms, and medical activity were almost identical and statistically significant. With regard to the analysis by type of pre-existing illnesses and type of severe acute symptoms (model 2), the predictors have been identical with the T3 results (respiratory, hormonal/metabolic diseases and dyspnoea, fatigue and concentration or memory problems). Cardiovascular illnesses and cough were also found to be statistically significant risk factors in the present study. In contrast, taste or smell disorders were not identified as a risk factor in the present follow-up analysis. The likelihood that medical activity is a surrogate for high socioeconomic status and therefore has a protective influence has already been discussed in Steinke et al. [10]. However, the consistency of these results was to be expected, as the recovery process in the cohort has hardly changed during the last follow-up and thus the data structure has also remained largely unchanged.

With regard to the published literature, gender and age are confirmed as relevant risk factors in various systematic reviews for persistent symptoms [16], [17], [18], [19]. Similar results can be observed in earlier studies for the number of previous illnesses as well as hormonal/metabolic and respiratory diseases [17], [18], [19]. The risk increase due to a higher number of severe acute symptoms during COVID-19 disease, which can be seen as a proxy for a severe acute course of the disease, can also be observed in various studies [19], [20].

With regard to the limitations of the study, it must be mentioned that the possibility of selection and recall bias cannot be ruled out. It was also not possible to compare the results with the prevalence of a proper control group from the general population. There is also the possibility that confounders that were not surveyed may have an influence on the results. The exclusion criterion of limited reading and writing skills and limited ability to speak German may also have led to exclusion from a PCS risk group. 

## Conclusions

Confirmatory results can be determined on the basis of this extended 4^th^ survey. It appears that minimal further recovery events can be expected in this cohort of insured persons after a 26-month follow-up. The extent to which these results on the prevalence of PCS can be explained by selection processes remains unclear. However, the identified risk factors, which remain stable over time, have largely been confirmed in the literature. The pragmatic gain in knowledge from this study to date is that COVID-19 patients with severe acute symptoms (especially cough, dyspnoea, fatigue and concentration or memory problems) should be taken seriously and monitored closely in the early stages of the disease. In combination with the other identified risk factors, it may be possible to identify high-risk individuals for PCS at an early stage and treat them appropriately in good time. 

## Notes

### Authors’ ORCIDs

- Nienhaus A: https://orcid.org/0000-0003-1881-7302
- Koch P: https://orcid.org/0000-0003-2865-9483

### Ethics approval

The study was conducted in accordance with the Declaration of Helsinki and approved by the Ethics Committee of the Hamburg Medical Association (protocol code 2021-10463-BO-ff, date of approval 16 March 2021). All participants gave their informed written consent to take part in the study.

### Funding

None.

### Availability of data and materials

The datasets used and analyzed during the current study are available from the corresponding author on reasonable request.

### Acknowledgements

We would like to thank all participants and everyone involved from the BGW and UKE, without whom this study would not have been possible.

### Competing interests

The authors declare that they have no competing interests.

## Figures and Tables

**Table 1:**
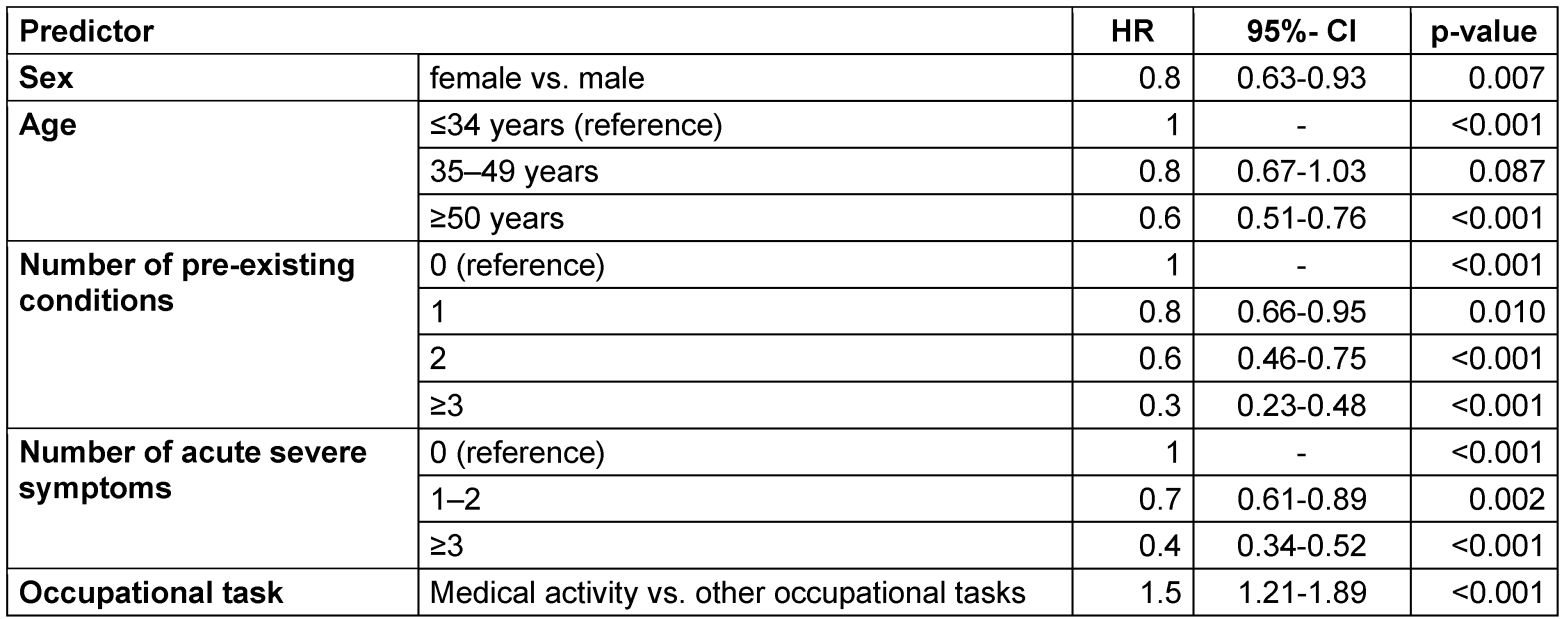
Multivariate Cox regression model 1

**Table 2:**
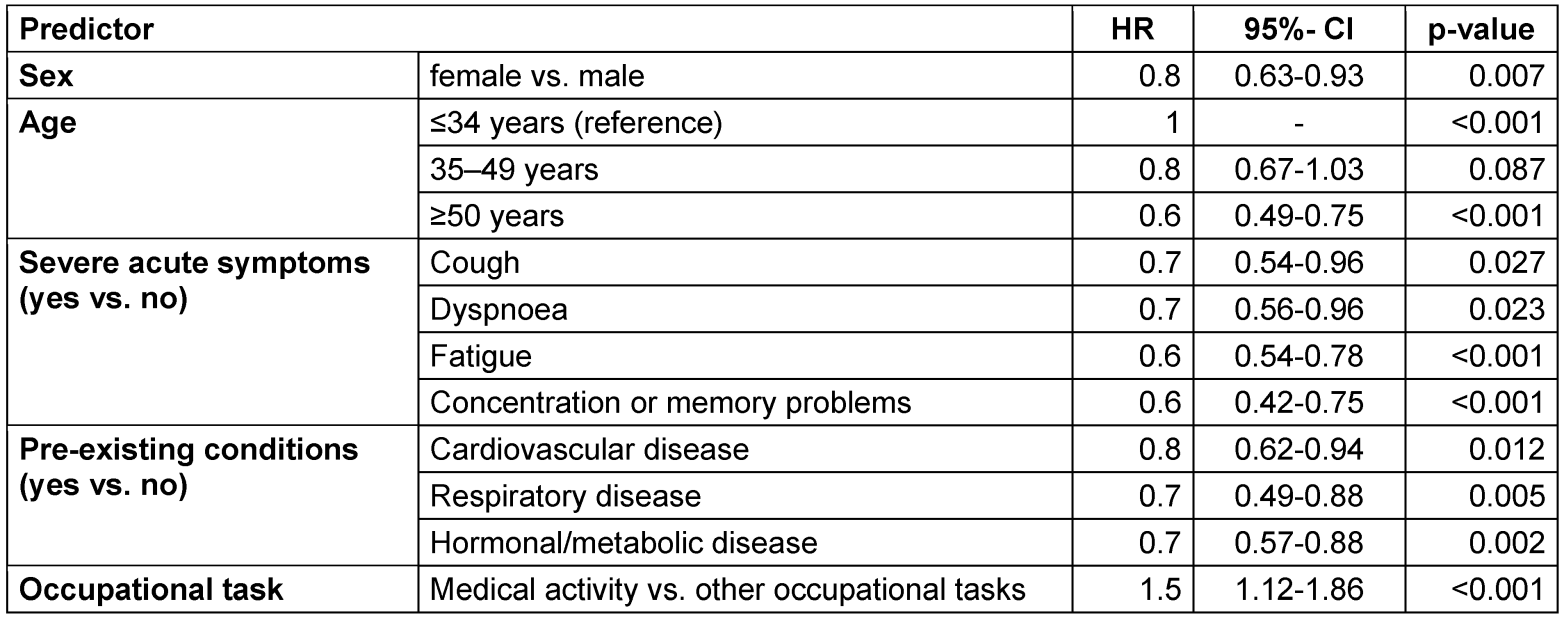
Multivariate Cox regression model 2
